# Expression of Ghrelin and Its Receptor mRNA in Bovine Oocyte and Cumulus Cells

**DOI:** 10.22074/ijfs.2019.5393

**Published:** 2018-10-02

**Authors:** Matías Angel Sirini, Juan Patricio Anchordoquy, Silvina Quintana, Cecilia Furnus, Alejandro Enrique Relling, Juan Mateo Anchordoquy

**Affiliations:** 1 IGEVET-Institute of Veterinary Genetic “Prof. Fernando N. Dulout” (UNLP-CONICET LA PLATA), Faculty of Veterinary Sciences, National University of La Plata, La Plata, Buenos Aires, Argentina; 2 Bee Reasearch Center, Department of Biology, FCEy N, National University of Mar del Plata - CONICET, Mar del Plata, Buenos Aires, Argentina

**Keywords:** Ghrelin, *GHS-R1A*, *In Vitro* Oocyte Maturation, mRNA Expression

## Abstract

Energy balance is regulated by ghrelin which is a neuroendocrine modulator. Ghrelin is expressed in repro- ductive organs. However, the role of ghrelin during *in vitro* maturation (IVM) and bovine preimplantational development is limited. The purpose of this study was to measure the expression of ghrelin (*GHRL*) and its receptor growth hormone secretagogue receptor 1A (*GHS-R1A*) mRNA, and determine cumulus oocyte complex (COC) viability after IVM with 0, 20, 40 and 60 pM of ghrelin. Also, pronuclear formation was recorded after *in vitro* fertilization (IVF). *GHRL* and *GHS-R1A* mRNA expression in oocyte and cumu- lus cells (CCs) was assessed using reverse transcription-polymerase chain reaction (PCR). Oocyte and CC viability were analyzed with the fluorescein diacetate fluorochrome-trypan blue technique. Pronuclear formation was determined 18 hours after IVF with Hoechst 33342. The results demonstrated that ghrelin mRNA is present in oocyte and CCs before and after 24 hours IVM with all treatments. Ghrelin receptor, *GHS-R1A*, was only detected in oocytes and CCs after 24 hours IVM with 20, 40 and 60 pM of ghrelin. Oocyte viability was not significantly different (P=0.77) among treatments. However, CC viability was significantly lower (P=0.04) when COCs were matured with ghrelin (77.65, 72.10, 66.32 and 46.86% for 0, 20, 40, and 60 pM of ghrelin, respectively). The chance of two pronuclei forming were higher (P=0.03) when ghrelin was not be added to the IVM medium. We found that ghrelin negatively impacts CC viability and pronuclear formation.

Nutrition has a strong influence on female bovine reproductive performance. In recent years, there has been a growing interest in investigating the relationship between nutrition and reproduction. In dairy cows, high milk yield leads to negative energy balance (NEB) which has adverse effects for fertility (1, 2). It has been suggested that metabolic hormones such as leptin and ghrelin might be signals that link fertility and energy status (3). Ghrelin is a neuroendocrine regulator of energy balance and food intake. Indeed, ghrelin plasma concentrations in cattle increase during fasting or NEB (4).

Previous studies have indicated that ghrelin regulates several reproductive functions (3, 4). Two subtypes of ghrelin receptors (GHS-R) have been identified, but only GHS-R type 1A (GHS-R1A) is functionally active (5). Recent investigations have localized ghrelin and *GHS-R1A* mRNA and protein expression to most reproductive tissues of dairy cattle (6). However, the expression of ghrelin and Received: 6/Sep/2017, Accepted: 10/Feb/2018 *Corresponding Address: IGEVET-Institute of Veterinary Genetic “Prof. Fernando N. Dulout” (UNLP-CONICET LA PLATA), Faculty of Veterinary Sciences, National University of La Plata, La Plata, Buenos Aires, Argentina Email: mateoanchordoquy@fcv.unlp.edu.ar Royan Institute International Journal of Fertility and Sterility Vol 12, No 4, Jan-Mar 2019, Pages: 335-338 its receptor in the bovine cumulus oocyte complex (COC) has not been yet described. Furthermore, the knowledge of ghrelin’s role in oocyte maturation and preimplantational development is very limited (4, 7-9). Therefore, the purpose of this study was to investigate ghrelin (*GHRL*) and *GHSR1A* mRNA expression in bovine oocyte and cumulus cells (CCs) after *in vitro* maturation (IVM) with different ghrelin concentrations, and evaluate the effect of ghrelin on oocyte and CC viability and pronuclear formation.

To perform this experimental research, bovine ovaries were obtained from an abattoir and transported to the laboratory in sterile NaCl solution (9 g/L) including the antibiotics streptomycin (100 mg/L) and penicillin (59 mg/L) at 37°C within 3 hours after slaughter. Ovaries were pooled, regardless of the estrous cycle stage of the donor. The COCs were aspirated from 3 to 8 mm follicles, using an 18-G needle connected to a sterile syringe. COCs with evenly granulated cytoplasms were selected under a low power (20-30 X) stereomicroscope (Nikon, Japan), and washed twice in TCM-199 buffered with 15 mM HEPES and IVM medium. Groups of 10 COCs were transferred into 50 μL of IVM medium under mineral oil (Squibb, USA). Incubation was performed at 39°C in an atmosphere of 5% CO_2_ in air with saturated humidity for 24 hours. COCs were matured in IVM medium supplemented with 0, 20, 40, and 60 pM acylated ghrelin. The total number of maturated COC was 1152. This total was divided on 200 COC for polymerase chain reaction (PCR) analysis, 480 for viability assay and 472 for pronuclear formation rates after *in vitro* fertilization (IVF).

After IVM, COCs were pipetted several times with a narrow-bore pipette in TCM-199 buffered with HEPES, and washed three times in calcium- and magnesium-free phosphate buffer solution (PBS) containing 1 mg/mL polyvinylpyrrolidone (PVP). Total RNA was isolated from CCs and oocytes with TRIzol (Invitrogen, CA) according to the manufacturer’s instructions. Samples were then treated with a RNase-Free DNase kit (Qiagen, Germany). The RNA content of each sample was calculated through 260 nm absorbance. RNA quality was evaluated by the ratio of absorbance at 260 and 280 nm with a NanoVue spectrophotometer (NanoVue™-NV-General Electrics Healthcare Limited, UK). Complementary DNA (cDNA) was synthesized using a reaction mixture containing 1.5 μg of total RNA, random hexamers and the M-MLV reverse transcriptase (Invitrogen-Life Technologies, USA), following the procedure suggested by the manufacturer. Polymerase chain reaction (PCR) was subsecquently performed on the cDNA from oocytes and CCs. The reaction were performed at a final volume of 25 μL containing 4 μL cDNA, 0.85 pmol/ mL of each primer, 0.2 mmol/L of each deoxynucleoside triphosphate, PCR buffer 1X (50 mmol/L KCl and 10 mmol/L TriseHCl, pH=8.3) and 0.1% Triton X-100, 1.2 mmol/L MgCl_2_, and 1.5 units of Taq DNA polymerase (Invitrogen, CA). The cDNA amplification reactions for (*GHRL*) and *GHS-R1A* were carried out with an initial denaturing step of 92°C for 3 minutes, followed by 35 cycles of 30 seconds at 92°C, 40 seconds at 60°C, and 40 seconds at 72°C, with a final elongation step of 72°C for 5 minutes. PCR products were verified on 2% agarose gel, stained with ethidium bromide, and visualized using a transilluminator with an UV filter. For the negative control, reverse transcription polymerase chain reaction (RT-PCR) procedures were carried out in the same manner, except that M-MLV reverse transcriptase was omitted during reverse transcription. The PCR reactions were performed in duplicates. Primers for each gene of interest were designed using Primer Premier Software (PREMIER Biosoft International, USA, Table 1), to avoid possible genomic DNA amplification, primers were designed to span exon-exon junctions. A total of 200 COCs were matured in two replicates (40 COCs per treatment). A time zero (T0, COC before IVM) treatment was used as the control group.

At the end of IVM, oocyte and CC viability were evaluated as follows. Oocytes were stripped of surrounding CCs by repeated pipetting in PBS containing 1 mg/mL PVP. Oocytes and CCs were incubated separately in the dark in 2.5 μg/L fluorescein diacetate fluorochrome and 2.5 g/L trypan blue in PBS medium for 10 minutes at 37°C. Then, they were washed three times in PBS. The CCs were centrifuge at 200 x g for 5 minutes. The pellet was resuspended in 50 μL of PBS. Oocytes and CC samples were transfered onto slides, which were immediately covered with cover slips and observed under a fluorescent microscope Olympus BX40 (Olympus, Japan) equipped with a 330-490 nm excitation filter and 420-520 nm emission filter. Live cells were visible with green fluorescence, whereas dead ones showed a characteristic blue staining under white light (Fig .1). A total of 480 COCs were matured in three replicates for this purpose.

**Fig.1:**
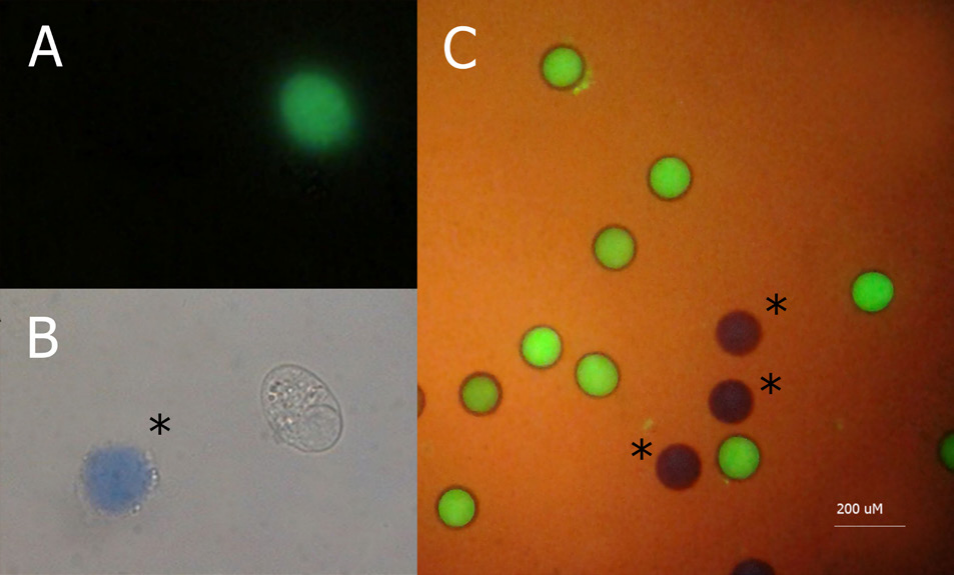
Oocyte and cumulus cells viability evaluated through fluorescein diacetate/trypan blue assay. The cell population was classified using the combined microscopic images obtained through light and fluorescence microscopy images. A. Alive cumulus cell (green) in the fluorescent field (×1,000 magnification), B. Dead cumulus cells (*) show a characteristic blue staining under white light (×1,000 magnification), and C. Alive (green) and dead (blue,*) bovine oocytes in a combined light and fluorescence field (×40 magnification).

The effect of different concentrations of ghrelin in the IVM medium on pronuclear formation was assessed after IVF (10). Expanded COCs were incubated in 50 μL of Fert-TALP under mineral oil. Frozen semen from A bull of the same strain was used in all experiments. Motile spermatozoa were separated by a discontinuous Percoll gradient. The final sperm concentration in the IVF medium was 2×106 spermatozoa/mL. Incubation was performed at 39°C and 5% CO_2_ in air with a saturated humidity for 18 hours. After IVF, presumptive zygotes were incubated in 0.1% (w/v) hyaluronidase in PBS solution for 5 minutes at 37ºC and then oocytes were denuded by gentle pipetting. The presumptive zygotes were incubated in 5 mg/L Hoechst 33342 in PBS for 30 minutes at 37ºC. Thereafter, they were examined under a fluorescent Olympus BX40 microscope (with a 365 nm excitation filter and a 400 nm emission filter) at ×200 and ×400 magnification to reveal the presence of pronuclei. A total number of 472 COCs were matured in three replicates for this purpose.

**Table 1:** Sequences of the primers for ghrelin (GHRL), growth hormone secretagogue receptor 1A (GHS-R1A) and the sizes of the reverse transcription polymerase chain reaction (RT-PCR) products


Gene	Primer sequence (5´-3´)	Temperature annealing (°C)	Amplicon size (pb)

*GHS-R1A*	F: ACAGACCGTGAAGATGCT	60	164
	R: GGTAGAAGAGGACGAAAGA	60	164
*GHRL*	F: CTGAAGAAA CCCTGGCTAAC	57	107
	R: CGTGGTCTCGGAAGTGTC	57	107


**Table 2:** Fertilization status of putative zygotes produced in vitro with various ghrelin concentrations in IVM medium


Treatments	Number of oocytes	n (%) 1 PN	n (%) 2 PN	n (%)>2 PN	n (%) penetrated

0 pM ghrelin	115	33^a^ (28.6)	69^a^ (60.0)	1^a^ (0.8)	103^a^ (89.5)
20 pM ghrelin	116	59^b^ (50.8)	48^b^ (41.3)	0^a^ (0)	107^a^ (92.2)
40 pM ghrelin	122	64^b^ (52.4)	45^b^ (36.8)	1^a^ (0.8)	110^a^ (90.1)
60 pM ghrelin	119	69^b^ (57.9)	46^b^ (38.6)	2^a^ (1.6)	109^a^ (91.5)


IVM; In vitro maturation, PN; Pronucleus, a, b; Within a column, values without a common superscript are significantly different (P<0.05), and COCs; Cumulus oocyte complex. Pronuclear rate was recorded 18 hours after insemination (472 COCs were matured and fertilized in three replicates). The presumptive zygotes were incubated in Hoechst 33342 and then examined under a fluorescent microscope at ×200 and ×400 magnification.

We used completely randomized block designs. Statistical models included the fixed effect of treatment (0 vs. 20 vs. 40 vs. 60 pM ghrelin) and the random effects of block (day of COCs collection, n=3). Oocyte and CC viability and rate of pronuclei presence were analyzed with logistic regression using the GENMOD procedure (SAS Institute, NC). Data for oocyte and CC viability and rate of pronuclei presence were expressed as a percentage. The level of significance was P≤0.05.

Using total RNA prepared from bovine oocytes and CCs and the specific primers for *GHRL, RT-PCR* showed a band of the expected size (107 bp) in agarose gel electrophoresis for all treatments (Fig .2). Thus, it seems clear that ghrelin mRNA is present in oocytes and CCs before and after 24 hours of IVM with 0, 20, 40 and 60 pM of ghrelin. On the other hand, the presence of *GHS-R1A* was only detected in oocytes and CCs after 24 hours of IVM with 20, 40 and 60 pM of ghrelin. The possibility of of contaminating genomic ghrelin and *GHS-R1A* sequence amplification was excluded since the band of the expected size was only detected in the presence of reverse transcriptase.

Oocyte viability was not significantly different (P=0.77) among COCs treated with 0, 20, 40, or 60 pM of ghrelin during IVM (89.0, 87.1, 88.0 and 89.1%, respectively). However, CC viability was significantly lower (P=0.04) in COCs matured with ghrelin (72.10, 66.32 and 46.86% for 20, 40, and 60 pM of ghrelin, respectively) than in COCs matured with 0 pM of ghrelin (77.65%). No differences were found between 20 and 40 pM of ghrelin. The lowest CC viability rate was observed with 60 pM of ghrelin (P=0.04).

The incidence of polyspermy (>2 pronuclei) and the percentage of mature oocytes penetrated by spermatozoa did not differ among treatments (P=0.96). However, the chance of two pronuclei forming (normal fertilization) were higher when ghrelin was not added to IVM medium (P= 0.03, Table 2).

**Fig.2:**
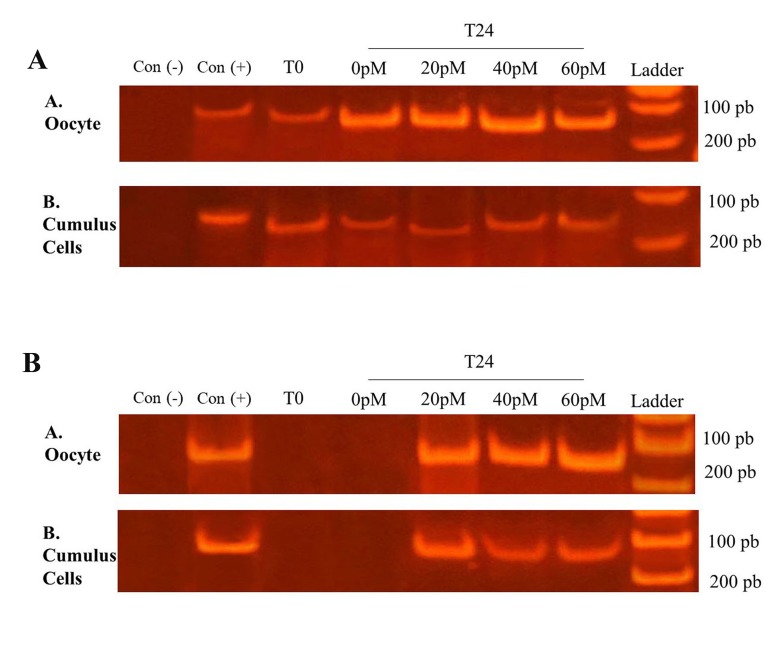
Agarose gel (2%) electrophoresis of polymerase chain reaction (PCR) products of *GHRL* and *GHS-R1A* cDNA. A. Agarose gel electrophoresis of PCR products of *GHRL* cDNA and B. Agarose gel electrophoresis of PCR products of *GHS-R1A* cDNA. COCs were matured 24 hours (T24) in IVM medium supplemented with 0, 20, 40, and 60 pM of ghrelin. A time zero (T0, COC before IVM) treatment was used. For the negative control [Con ()], RT-PCR procedures were carried out in the same manner, except that M-MLV reverse transcriptase was omitted during reverse transcription. Hypothalamus tissue was used as a known positive control sample [Con (+)].

To our knowledge, this is the first study to report the expression of ghrelin and its receptor *GHS-R1A* in bovine oocytes and CCs. Our results indicate that ghrelin mRNA expression can be detected in oocytes and CCs both before and after IVM regardless of ghrelin presence during the IVM process. These findings support the idea that ghrelin may have an autocrine and/or paracrine effect within the follicular microenvironment. On the other hand,* GHS-R1A* mRNA expression was only detected when ghrelin was added to the IVM media, suggesting that the presence of ghrelin in the environment surrounding COCs may stimulate the expression of its functional receptor in both bovine oocytes and CCs. It has been demonstrated that ghrelin increases *GHS-R* mRNA levels in rat neurons (11). The mRNA expression of *GHS-R1A* is regulated by endogenous agonists, hormones and transcriptional factors (TFs) (12, 13). One of these factors, the pituitary-specific transcription factor (POU1F1) increases the expression of *GHS-R1A* and is present in oocytes and preimplantational embryos (13-15). García et al. (16) demonstrated that ghrelin induces the activation of Pit-1 (POU1F1) in anterior pituitary cells of infants. Although, in this study we did not examine the expression of Pit-1 in bovine COCs, this TF could have increased expression in the presence of ghrelin during IVM.

Cumulus cells play a key role in the acquisition of nuclear and cytoplasmic oocyte maturation (17). Furthermore, CCs protect the oocyte against oxidative stress and apoptosis (18). Likewise, CC damage leads to both lower fertilization and blastocyst formation rates (19, 20). In the present study, bovine COCs matured with different ghrelin concentrations resulted in a reduction of CC viability. Also, normal fertilization (formation of two pronuclei) was affected when oocytes were matured *in vitro* in the presence of ghrelin. Even though, the information about the effect of ghrelin on oocyte maturation and early embryo development is scarce and contradictory. However, our findings about the negative effect of ghrelin are in agreement with several publications (4, 8, 9).
